# Supplementary material for: Protein Supplementation with Short Peptides Prevents Early Muscle Mass Loss after Roux-en-Y-Gastric Bypass
Source: Nutrients. 2022 Dec 1;14(23):5095. doi: 10.3390/nu14235095 (PMC9736680; doi:10.3390/nu14235095)
Supplement: Supplementary file 1 [file nutrients-14-05095-s001.zip › nutrients-2059481-supplementary.pdf]

<u>Valoración organoléptica del suplemento nutricional (SPANISH)</u>

Cumplimiento del suplemento nutricional (batido)

□ 0% □ 25% □ 50% □ 75% □ 100%

En caso de no haber cumplido el 100% de la toma indique el motivo de interrupción:

¿Le ha gustado el sabor del batido?

□ Sí □ No

¿Le ha gustado el olor del batido?

□ Sí □ No

¿Le ha gustado el color del batido?

□ Sí □ No

¿Le ha saciado el batido? (marca según el nivel de saciedad)

□No (0%) □Un poco (25%) □Normal (50%) □Bastante (75%) □Mucho (100%)

¿Ha tolerado el batido?

□ Sí □ No

¿Ha presentado algún efecto secundario?

□ Sí □ No

En caso afirmativo, marque los efectos secundarios:

- □ Vómitos
- □ Náuseas
- □ Mareos
- □ Dolor abdominal
- □ Diarrea

¿Qué cambiaría del producto nutricional?

<u>Organoleptic evaluation of the nutritional suplement (ENGLISH)</u>

Compliance with the nutritional supplement (shake)

□ 0% □ 25% □ 50% □ 75% □ 100%

If you have not completed 100% of the intake, indicate the reason for interruption:

Did you like the taste of the smoothie?

□ Yes □ No

Did you like the smell of the smoothie?

□ Yes □ No

Did you like the colour of the smoothie?

□ Yes □ No

Has the milkshake filled you up? (mark according to the level of satiety)

□No (0%) □A little (25%) □Normal (50%) □Quite a bit (75%) □A lot (100%)

Have you tolerated the shake?

□ Yes □ No

Have you had any side effects?

□ Yes □ No

If yes, please check the side effects:

□ Vomiting
□ Nausea
□ Dizziness
□ Abdominal pain
□ Diarrhea

What would change the nutritional product?
